# HCMV detection in Asian gastric cancer RNA-seq data sets and clinical validation in Indian GC patients reveals the HCMV-GC specific gene signatures

**DOI:** 10.1128/msystems.00673-24

**Published:** 2024-09-16

**Authors:** Pandikannan Krishnamoorthy, Athira S. Raj, Nilanjana Das, Saia Chenkual, Jeremy L. Pautu, Lalengkimi Ralte, Nachimuthu Senthil Kumar, Himanshu Kumar

**Affiliations:** 1 Department of Biological Sciences, Laboratory of Immunology and Infectious Disease Biology, Indian Institute of Science Education and Research (IISER) Bhopal, Bhopal, Madhya Pradesh, India; 2 Department of Surgery, Zoram Medical College, Falkawn, Mizoram, India; 3 Department of Medical Oncology, Mizoram State Cancer Institute, Zemabawk, Mizoram, India; 4 Department of Biotechnology, Mizoram University, Aizawl, Mizoram, India; 5 Laboratory of Host Defense, WPI Immunology, Frontier Research Centre, Osaka University, Osaka, Japan; Argonne National Laboratory, Lemont, Illinois, USA

**Keywords:** gastric cancer, HCMV, meta-analysis, RNA-seq, clinical screening

## Abstract

Gastric cancer (GC) prevalence is very high in the Asian population. Oncogenic viruses play a crucial role in the progression of different types of cancers. Through reanalysis of clinical RNA-seq data sets derived from Asian GC patients, this study identified the presence of human cytomegalovirus (HCMV) in Asian GC tumors, next to the well-studied association of EBV. Clinical recruitment of the Indian GC cohort and screening for HCMV presence identified a 14.28% occurrence, similar to that observed in the bioinformatics analysis. A combinatorial approach of rank-based meta-analysis and ranking of groups based on an expectation-maximization algorithm identified that the upregulated LINC02864 and MAGEA10 correlated with poor survival of GC patients and downregulated tumor suppressor genes enriching for gastric acid secretion pathway to be associated with HCMV-positive GC patients, revealing the progressive role of HCMV infection in GC. Genes that discriminate between different stages of GC were identified through feature selection implemented in a machine-learning approach. LTF and KLK10 expressions were found to be specifically dysregulated by HCMV and can also indicate the GC stages. The results of this study will guide future studies to identify the functional role of these genes in the HCMV-associated GC.

**IMPORTANCE:**

Nearly 75% of gastric cancer (GC) cases reported globally are from the Asian population. Most existing public databases, such as TCGA, comprise only a fractional portion of data derived from Asian ancestry. This study identified EBV and human cytomegalovirus (HCMV)'s higher detection in GC patients. The presence and role of EBV associated with GC are well-known, and the observation of HCMV prompted us to validate the findings in a small cohort of 40 Indian GC patients. We observed a 14.28% occurrence of HCMV in the Indian cohort, similar to that observed from next-generation sequencing. A combinatorial approach of rank-based meta-analysis and ranking of groups based on an expectation-maximization algorithm identified that the upregulated LINC02864 and MAGEA10 correlated with poor survival of GC patients and downregulated tumor suppressor genes enriching for gastric acid secretion pathway to be associated with HCMV-positive GC patients, revealing the progressive role of HCMV infection in GC.

## INTRODUCTION

Gastric cancer (GC), or stomach cancer, ranked fifth in incidence and fourth in mortality among all other cancers in 2020. Almost 75% of the total GC cases and GC mortality globally reported were observed in the Asian population (1). GC development/progression is multifactorial and associated with genetic and environmental risk factors. The common risk factors of GC include *Helicobacter pylori* infection, genetics, smoking, excessive consumption of red meat and alcohol, obesity, low socioeconomic status, and, to some extent, blood group, surgery, radiation, ethnicity, and sex (2). The prognosis and 5-year survival rate of GC also remain low in Asian populations (1).

Over time, it has been widely recognized that some virus infections are linked to many cancers. Human herpes viruses (HHV) and human papillomavirus (HPV) were reported to play a crucial role in the tumor progression in many types of cancers (3). The association of GC with Epstein-Barr virus (EBV), also denoted as human herpesvirus-4 (HHV-4), has been explored by many individual studies (4). Apart from EBV, several epidemiological and seroprevalence studies identified the presence of human cytomegalovirus (HCMV), hepatitis B virus (HBV), and HPV in GC samples (5). HCMV, also denoted as HHV-5, was well-studied for its association with tumor pathogenesis in many cancers. Many independent studies reported the presence of HCMV in GC patients, but all these were either epidemiological or seroprevalence studies and were qualitative. Moreover, these studies are quite contradicting in nature, with few studies showing HCMV to be positively correlated while others show its negative correlation with GC progression (6–8). Moreover, the transcriptome responses associated with HCMV infection GC remain unexplored.

This study aims to screen for 762 viruses in the Asian GC patient samples by extracting the human unmapped reads and aligning them to the virus genomes. Apart from the well-known association of EBV, the presence of HCMV in GC was identified in three independent patient data sets from China and Korea. Clinical validation of the presence of HCMV in Indian GC patients was performed. Using the combinatorial approach of rank-based meta-analysis, network, and machine learning algorithms, HCMV GC-specific gene signatures associated with different stages of GC were identified.

## MATERIALS AND METHODS

### Recruitment of GC patients and collection of demographic and clinical metadata

Tumor tissues from GC patients were collected from the Mizoram State Cancer Institute, Zemabawk, Aizawl, and Civil Hospital Aizawl, Mizoram, India, between August 2019 and March 2020. Forty-two surgically operated GC biopsy samples and adjacent healthy tissues from patients were collected based on prior consent. GC patients with other chronic diseases, past history, or present record of gastritis treated for any other types of cancer were excluded from the cohort. All GC tissue samples were confirmed histopathologically. All volunteers were fully informed about the study and participated with their full consent. The demographic details of all the samples involved in the study are tabulated in Table S1.

### Screening of HCMV-positive GC patients using nested PCR

DNA isolation from the tissue samples was done using Qiagen DNeasy Blood & Tissue Kit following the manufacturer’s protocol. The highly conserved immediate early gene (IE gene) of HCMV was targeted for PCR amplification. The nested PCR method was performed to screen the samples to avoid any false positive results. The primers OF_HCMV (5′-CATGATTGCGGGTGTAGATG-3′) and OR_HCMV (5′-CCCCTCATCAAACAGGAAGA-3′) targeted the outer region, while the primers IF_HCMV (5′-GGGATCATGAATGGCAGATT-3′) and IR_HCMV (5′-TTCGGACTCGGAGAGTGAGT-3′) targeted the inner region of the IE gene. To further ensure our observations, the samples detected positive in the nested PCR method were subjected to Sanger sequencing, and the sequence obtained was queried in the BLAST tool to identify the presence of HCMV.

Adjacent normal tissues were also subjected to the PCR analysis along with tumor samples under the same condition and were all found to be negative.

### Asian GC transcriptome data selection and viral read quantification

The bulk-RNA sequencing transcriptome data sets were searched and retrieved from the Gene Expression Omnibus database so that only the data sets with a minimum of 50 tumor samples derived from the tumor tissue samples of Asian GC patients were sequenced. As per preferred reporting items for systematic reviews and meta‐analyses guidelines, the eligible data sets were identified as explained in (Fig. S1). The raw fastq files of all the GC tumor samples were downloaded from the Sequence Read Archive database. VIRTUS2 (VIRal Transcript Usage Sensor) pipeline was implemented to quantify the viral reads with default parameters (9). The quality-checked, adapter-trimmed fastq files were aligned to the human genome (hg38) using STAR (10). The unmapped reads were aligned to 762 viruses and processed further to quantify the viral transcripts present in the clinical samples, as documented in the VIRTUS2 pipeline.

Once the number of reads mapped to 762 viral genomes across all samples was quantified, the aim was to identify the viral genomes that had a higher number of reads mapped in most of the samples across all data sets. There could be technical artifacts while sequencing or possible contamination that could give us false-positive reads interpretation. To avoid such possibilities, screening was performed for the viruses that were detected in most of the samples in each data set. There are certain cases where only few reads were detected for viral genomes in a few samples, and those samples were not qualified for further study. If the viral genomes are not detected in more than one data set, there is a possibility that those could be contaminations. Overall, the highly confident viral genomes were detected in most of the samples and have a higher amount of reads and were detected in all three data sets. In order to avoid the possibility of attaining false-positive results regarding the number of reads mapped to the viral genomes due to varied library sizes in each sample, reads mapped on viral genome/read mapped on human genome (V/H ratio) were calculated.

In each data set, viruses with a minimum of 100 reads in at least one sample and a total of 1,000 viral reads across the total number of samples were plotted as the heatmap. The number of viral reads across each sample in every data set and the details regarding the selection of the top prevalent viruses in each GC tumor sample are provided in Table S2.

### Meta-analysis to identify the top genes associated with HCMV-associated GC using rank aggregation

The aligned reads (binary alignment files) obtained after the alignment with STAR in each eligible data set were quantified for the host gene expression using featureCounts (11). Raw counts were processed, and differential expression analysis between HCMVPosGC (the samples with minimum 10 of HCMV viral reads and no reads for EBV) and HCMVNegGC (the samples with no reads detected for HCMV and EBV) was performed in each data set to identify the significant genes dysregulated using DESeq2 in all three data sets (12). Pathway enrichment analysis was performed using the R package clusterProfiler (13), and dot plots were used to visualize the top significant pathways enriched for significant upregulated and downregulated genes in each data set. The circos plot and the chord plot were plotted through the GOplot package after performing reactome pathway enrichment analysis through the DAVID tool (14, 15). Rank-based meta-analysis was performed using the package RobustRankaggreg (RRA) to identify the top significant genes consistently dysregulated in each data set (16). Heatmap was used to visualize the genes with adjusted *P* value e^−2^ and logFC of at least 1 in each data set and −2.5 > logFC > 2.5 after RRA implementation. Survival analysis, along with the expression of the top most upregulated genes, was performed using the web tool SurvivalGenie, which uses the survival information of stomach adenocarcinoma patients derived from the TCGA database (17).

### RNA isolation and quantitative PCR

Tissue samples were homogenized and total RNA was isolated using TRIzol reagent (Ambion/Invitrogen). cDNA was synthesized using iScript cDNA synthesis kit (Bio-Rad) following the manufacturer’s protocol. Gene expression was estimated by quantitative real-time PCR using SYBR green chemistry (Kappa) and gene-specific primers GAPDH (5′-ATCATCCCTGCCTCTACTGG-3′, 5′-GTCAGGTCCACCACTGACAC-3′), Linc02864 (5′-CATGGGAATGGACACAGGG-3′, 5`-CGCCACTGTAGGGACATCAT-3′), and MAGEA10 (5′-CAATCCCAAAGTGAGACACAGG-3′, 5′-GGGGTGCTTGGTATTAGAGGA-3′).

### Ranking algorithm based on posterior probability to identify HCMVGC-specific genes

Due to higher sample size, GSE184336 data set is segregated into three groups of GC patients: HCMV_GC GC samples with minimum of 10 HCMV reads and no EBV reads; HCMV_EBV_GC, GC samples with minimum cumulative sum of 10 reads of HCMV and EBV and presence of both virus reads; and EBV_GC, GC samples with minimum of 10 EBV reads but no HCMV reads. The raw counts were normalized and the segregation of the groups was visualized through the PCA plot. Since this multi-class problem and the motive is to identify the HCMV_GC specific gene signatures, the geNetClassifier R package was implemented to rank the genes with the greatest discriminant power in an unbiased manner (18). The top 50 genes that exceeded the posterior probability cutoff (>0.95), as per default value suggested in the package, were used to construct gene correlation and interaction network.

### Feature selection to find the GC stage discriminatory genes

GSE184336 data set only has the information of stages of each patient, and they were classified into stage I, stage II, stage III, and stage IV. After normalization, removal of low expressed genes, and sample outlier detection, a set of genes that can discriminate different stages of GC was identified through the DaMiRSeq R package as performed in the previous study (19, 20). The clustering of different classes of highly significant features was visualized through multi-dimensional scaling (MDS). The top significant features were ranked based on the importance of a gene with better classification by computing a score called RReliefF score. The expression of selected genes in single-cell RNA-sequencing data set GSE134520 was plotted using the TISCH2 (21).

## RESULTS

### Detection of viral genomes in Asian GC patients

**Fig 1:**
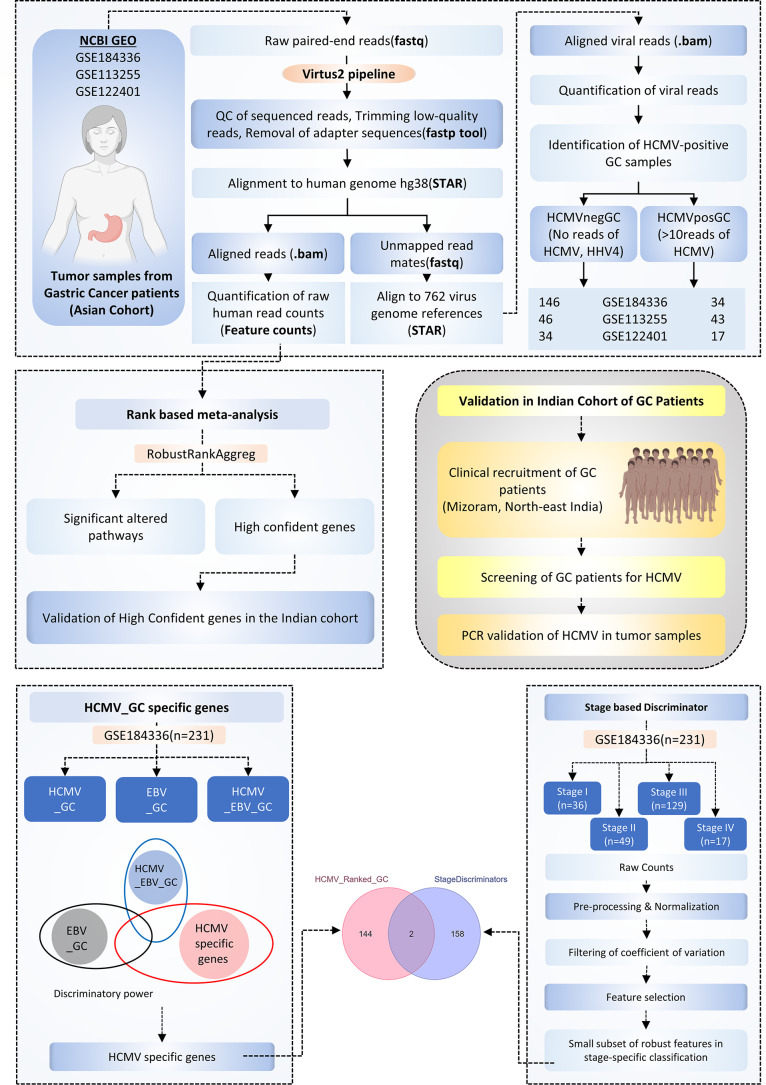
Workflow for identifying HCMV detection and associated gene signature in Asian GC patients. (**A**) The Schematic of the pipeline used to quantify the viral reads from the RNA-seq data sets. Raw fastq files were quality checked, adapter trimmed, and aligned to the human genome. The mapped reads were used to quantify the host gene expression, while the unmapped reads were aligned to 762 virus genomes to quantify the viral reads present in each sample using the VIRTUS2 pipeline. Tumor samples from 42 Indian GC patients were subjected to nested PCR to identify the presence of HCMV samples. Rank-based meta-analysis identifies genes associated with HCMVPosGC and gene ranking based on posterior probability identifies HCMV_GC specific genes and the final part involves the identification of genes associated with HCMV_GC and different stages of GC progression.

**Fig 2:**
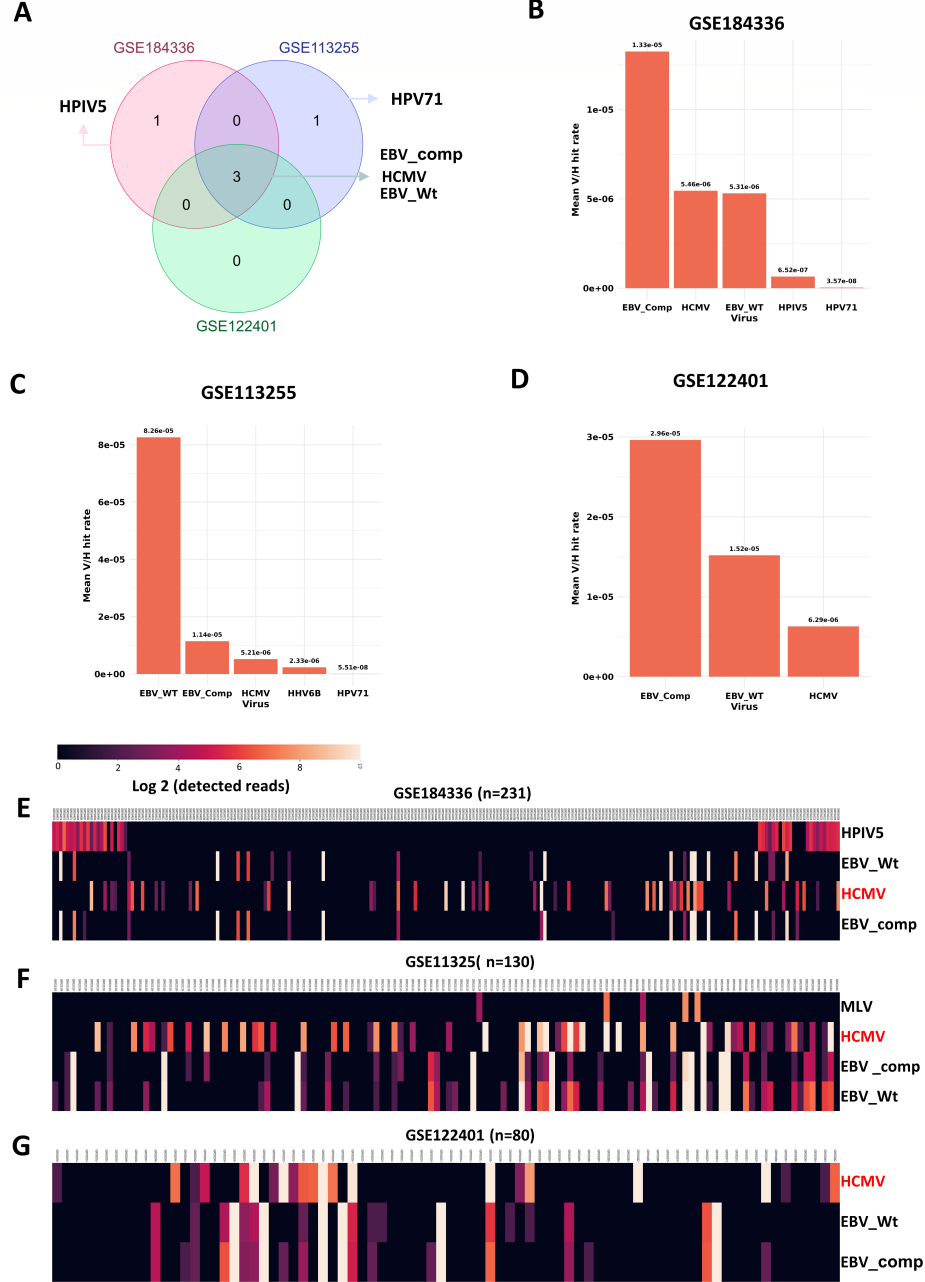
Screening of viruses from high-throughput sequencing data sets derived from Asian GC patients. (**A**) Venn diagram represents the top viruses that are detected in more than 10 samples and 10 reads in the data sets GSE184336, GSE113255, and GSE122401. EBV and HCMV are the common viruses detected in all three data sets, while HPIV5 and HPV71 are detected only in GSE184336 and GSE113255, respectively. The mean value of V/H ratio, reads mapped on viral genome/read mapped on human genome of all samples of top viruses are represented as bar plots for the data sets (**B**) GSE184436, (**C**) GSE113255, and (**D**) GSE122401. Heatmap denoting the viruses with a minimum of 100 reads in at least one sample and a total of 1,000 reads across all the samples in the data sets, (**E**) GSE184336, (**F**) GSE113255, and (**G**) GSE122401. HPIV5. human parainfluenza virus 5; HCMV, human cytomegalovirus; EBV-4_wt, Epstein-Barr virus wild-type genome; HHV-4_com, human herpes virus-4 complete genome; and MLV, murine leukemia virus.

Three eligible raw RNA sequencing data sets were selected to screen for the presence of 762 virus sequences in Asian gastric cancer patients (Fig. S1). The data sets, GSE184336, GSE113255, and GSE122401, included tumor samples from 231 Chinese GC patients, 130 South Korean GC patients, and 80 South Korean GC patients, respectively (Fig. 1) (22–24). After aligning the quality-checked and adapter-trimmed raw reads with the human genome, the unmapped reads were aligned to 762 different virus genomes, as implicated in the VIRTUS2 pipeline (Fig. 1) (9). The total number of reads aligned to every viral genome in all three data sets is provided in Table S2. There could be possibility of false-positive results due to technical artifacts or contaminations in fewer samples. To reduce such possibility, screening was performed in such a way that virus is considered to be detected only if minimum of 10 reads are present in the sample and detected in minimum of 10 samples in each data set. A total of five viral genomes were detected in higher number of samples across all three data sets (Fig. 2A). Human parainfluenza virus (HPIV5) was detected in 35 samples in GSE184336 data set alone but not in other data sets, while human papilloma virus-71 (HPV71) was detected in 23 samples in GSE113255 but not in other data sets. HHV4 (EBV)_Wildtype genome and EBV_Complete genome belonging to EBV and HHV5 (HCMV) genome are the three viral genomes detected in most samples in all three data sets (Fig. 2A). Further, reads mapped on viral genome/read mapped on human genome (V/H ratio) for each sample in every data set for all viral genomes was calculated. This approach of identifying the V/H ratio of viral genomes normalized to the human mapped reads is to ensure that the detection is not due to the library bias or technical artifacts. EBV and HCMV are the top viruses whose V/H ratio did not go less than 10^−6^ in the data sets GSE184336 (Fig. 2B), GSE113255 (Fig. 2C), and GSE122401 (Fig. 2D), respectively. The heatmap denoting the viruses whose cumulative read counts were 1,000 across all the samples in each data set also denotes the presence of EBV and HCMV to be the top viral genomes present in the Asian GC samples in the data sets GSE184336 (Fig. 2E), GSE113255 (Fig. 2F), and GSE122401 (Fig. 2G), respectively. HPIV5 was detected only in one data set and that too in a batch of samples as denoted in the bubble plot (Fig. S2D), which could be due to the contamination while processing those samples, as they are known to be contaminants of tissue samples. HHV7, MLV, XMRV, and HHV6 are detected in very few samples and hence the possibility of their functional association may be minimal. The aligned viral reads mostly map for the four well-known non-coding RNAs derived from HCMV—RNA2.7, RNA1.2, RNA4.9, and RNA5.0. RNA2.7 is highly detected in all three data sets (Fig. S3).

Through three different approaches screening for 10 reads in minimum of 10 samples across three data sets through the Venn diagram (Fig. S2A through C), top viruses with higher V/H ratio in every data set and heatmap plotting the viral genomes with cumulative read counts of 1,000 in each data set, it is evident that EBV and HCMV are the top viruses present in the Asian GC tumor samples. HCMV was detected in 34 out of 231 GC samples, 43 out of 130 GC samples, and 17 out of 80 GC samples in the data sets GSE184336, GSE113255, and GSE122401, respectively. Although the association of EBV with the GC is well-studied, the association of HCMV with GC and their functional relevance are not explored. Hence, we focused more on HMCV and how it affects the host transcriptome response in GC patients.

### Detection of HCMV infection in Indian GC patients

**Fig 3:**
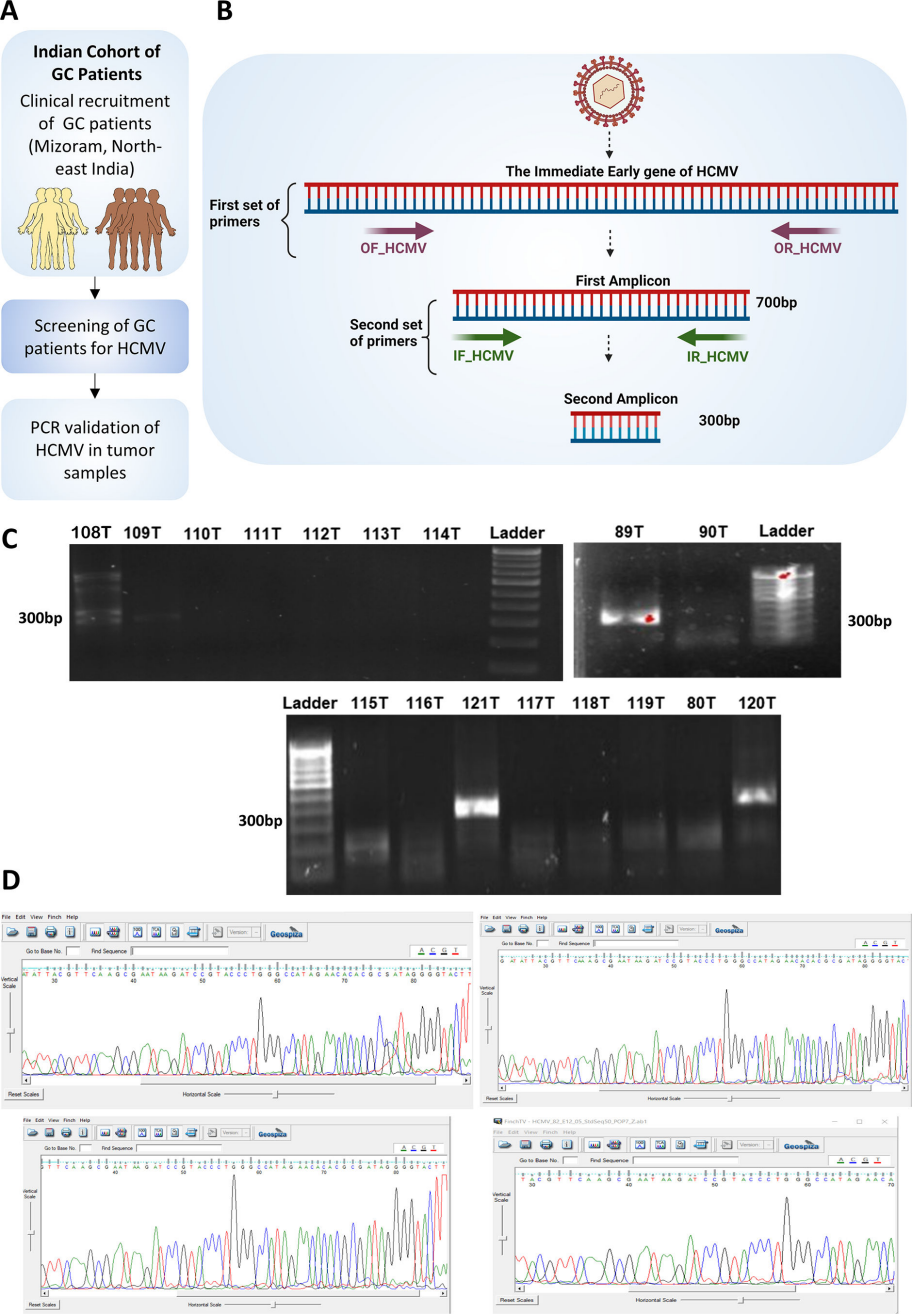
Screening of HCMV in Indian GC patients using nested PCR. (**A**) GC tumor tissues were collected from 42 GC patients from Mizoram, India, where GC is highly prevalent. (**B**) To avoid false-positive detection, nested PCR targeting the immediate early gene of HCMV was performed. (**C**) Nested PCR identified the presence of HCMV in the clinical samples visualized by agarose gel electrophoresis. (**D**) Sanger sequencing was performed to confirm the presence of HCMV in the clinical samples.

The presence of HCMV reads observed in RNA-seq data sets of Asian GC samples was further validated in the clinical cohort from Mizoram, where GC cases were high in India (25). A total of 42 GC tissue biopsy samples were recruited, and demographic details were collected for each of the patients enrolled in the study (Fig. 3A). Nested PCR targeted the highly conserved immediate early (IE) gene of HCMV to screen for GC samples with HCMV infection (Fig. 3B). PCR amplification occurred in 6 out of 42 GC samples enrolled in the study (Fig. 3C). Sanger sequencing of the PCR products from the six GC samples was performed to further verify the presence of HCMV sequences in these samples (Fig. 3D). The frequency of HCMV detection in RNA-seq samples was 14.7%, 33%, and 21.2% in the data sets GSE184336, GSE113255, and GSE122401. 14.28% HCMV detection was found in the Indian GC patients.

### Differential expression analysis between the HCMV-positive GC and HCMV-negative GC identifies potential pathways

**Fig 4:**
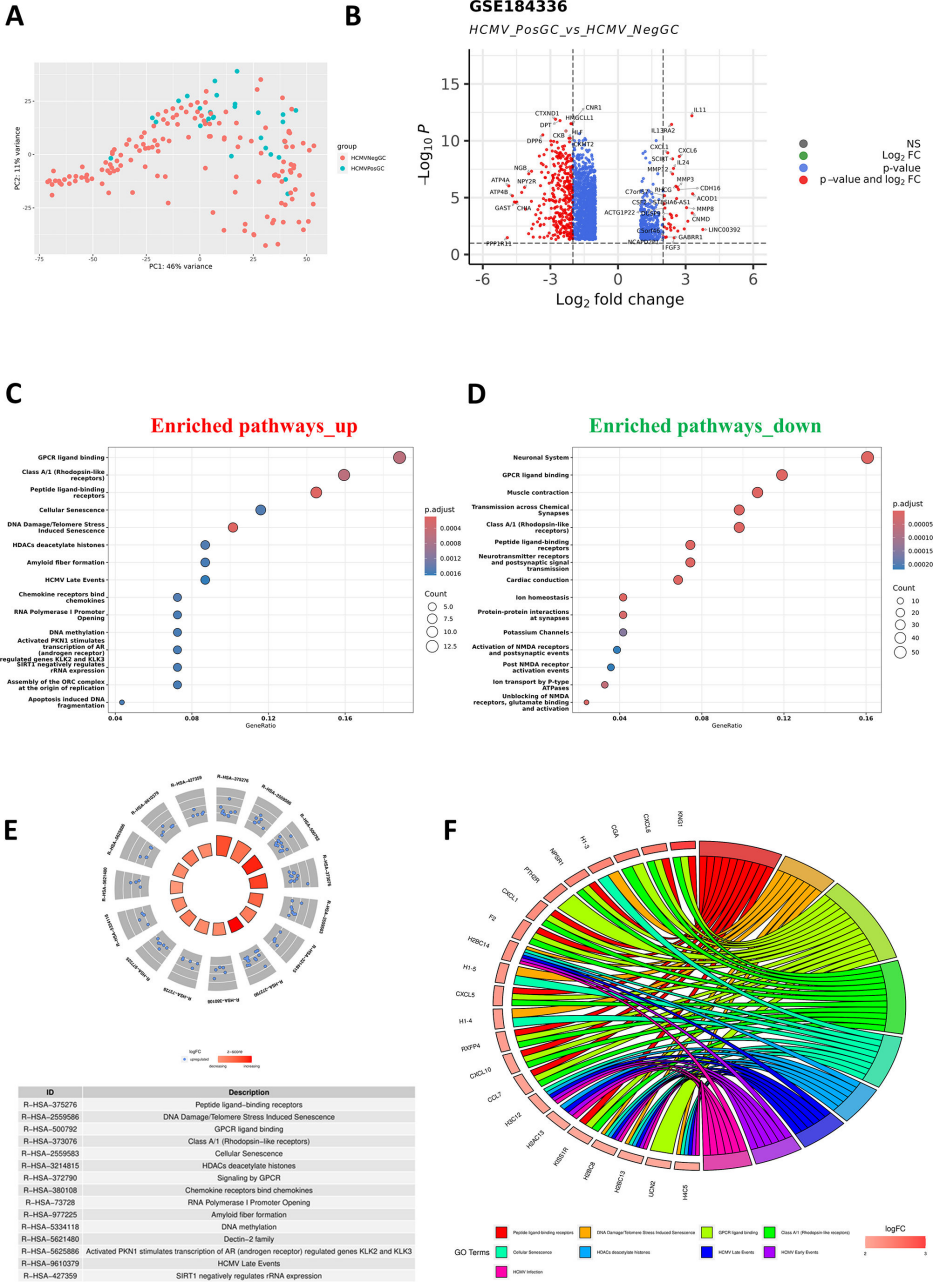
Significant genes enriched between HCMVPosGC and HCMVNegGC in the GSE184336 data set. (**A**) Differential expression analysis between HCMVPosGC (samples with more than 10 HCMV reads) and HCMVNegGC (samples with no reads of EBV and HCMV) was performed. PCA plot depicting the segregation of the groups. (**B**) Significantly upregulated and downregulated genes were visualized through a volcano plot. (**C**) Over-representation analysis for reactome pathways for upregulated and (**D**) downregulated genes were represented in dot plots. (**E**) Significant pathways dysregulated and the log-fold change of associated genes were visualized through the circos plot. (**F**) The relation between the top genes and pathways was represented through the chord plot.

To identify the transcriptome response associated with HCMV infection in GC patients, only the samples with no reads for both the HCMV and EBV were categorized as HCMV negative GC (HCMVNegGC) group. The presence of a minimum of 10 HCMV reads in the sample was categorized as HCMV positive GC (HCMVPosGC) group (Fig. 4A). Differential expression analysis between both these groups identified 1,839 genes to be significantly dysregulated with a significance threshold of −1 > logFC > 1 and *P*adj < 0.05 in the data set GSE184336 (Fig. 4B; Fig. S4B and C). Over-representation analysis for the reactome pathway identified pathways such as GPCR ligand binding, HDACs deacetylase histones, and HCMV_late events to be enriched for the upregulated genes (Fig. 4C), while pathways such as neuronal system, GPCR ligand binding, Muscle contraction to be enriched for the downregulated genes (Fig. 4D). Circos plot denotes the top pathways that are dysregulated along with the logFC values of associated genes (Fig. 4E), and the chord plot denotes the top pathways with their associated genes (Fig. 4F). It has been observed that the pathways such as GPCR ligand binding, HCMV_late event genes are upregulated in the HCMV-positive GC patients compared to HCMV-negative GC patients.

### Meta-analysis to identify the significant genes associated with HCMV-associated GC using rank aggregation

**Fig 5:**
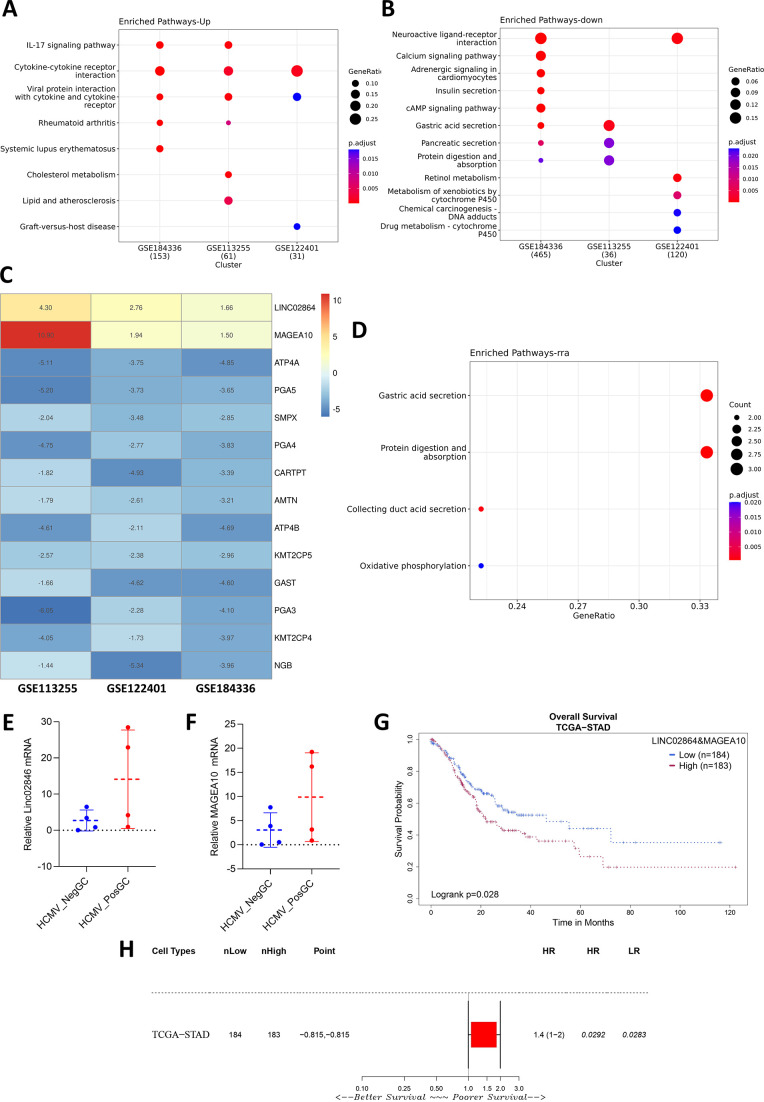
Rank-based meta-analysis identified the robust genes associated with HCMV-positive GC. (**A**) Differential expression analysis and over-representation analysis of the upregulated and (**B**) downregulated genes in all three data sets were visualized through the dot plot. (**C**) Heatmap representing the logFC of the robust genes in each data set. (**D**) Dotplot depicting the pathways enriched for the top robust genes identified through robust rank aggregation. Expression of top robust upregulated genes (**E**) LINC02864 and (**F**) MAGEA10 in the Indian GC cohort using RT-PCR. (**G and H**) Survival analysis identified that overexpression of these genes is associated with poor survival outcomes.

Differential expression analysis between HCMVPosGC and HCMVNegGC groups identified 240 genes and 401 genes to be significantly dysregulated, with a significance threshold of −1 > logFC > 1 and *P*adj <0.05, in the data sets GSE113255 and GSE122401, respectively (Fig. S4B and C; Table S3). Pathway enrichment analysis of all data sets identified pathways such as cytokine-cytokine receptor interaction to be enriched for the upregulated genes (Fig. 5A) and pathways such as gastric acid secretion, Neuroactive ligand interaction to be enriched for the downregulated genes (Fig. 5B).

In order to identify the consistently dysregulated high-confidence genes associated with HCMV-positive GC, the robust rank aggregation method was performed. The high-confidence genes and logFC values in each data set were visualized using a heatmap (Fig. 5C; Table S4). Significantly downregulated genes were majorly involved in the gastric acid secretion pathway and protein digestion absorption pathway that was associated with GC (Fig. 5D). Two genes were consistently upregulated in the HCMV-positive GC patients. LINC02864 and MAGEA10 (melanoma-associated antigen 10) were consistently upregulated in the HCMVPosGC group. These two genes were also observed to be upregulated significantly in the HCMV-positive Indian GC patient samples (Fig. 5E and F). Survival analysis was performed over stomach adenocarcinoma (STAD) patients' data in TCGA, and it was observed that these two genes' upregulation leads to poorer survival outcomes in STAD patients (Fig. 5G and H).

### Identification of HCMVGC-specific signature and network based on discriminatory power

**Fig 6:**
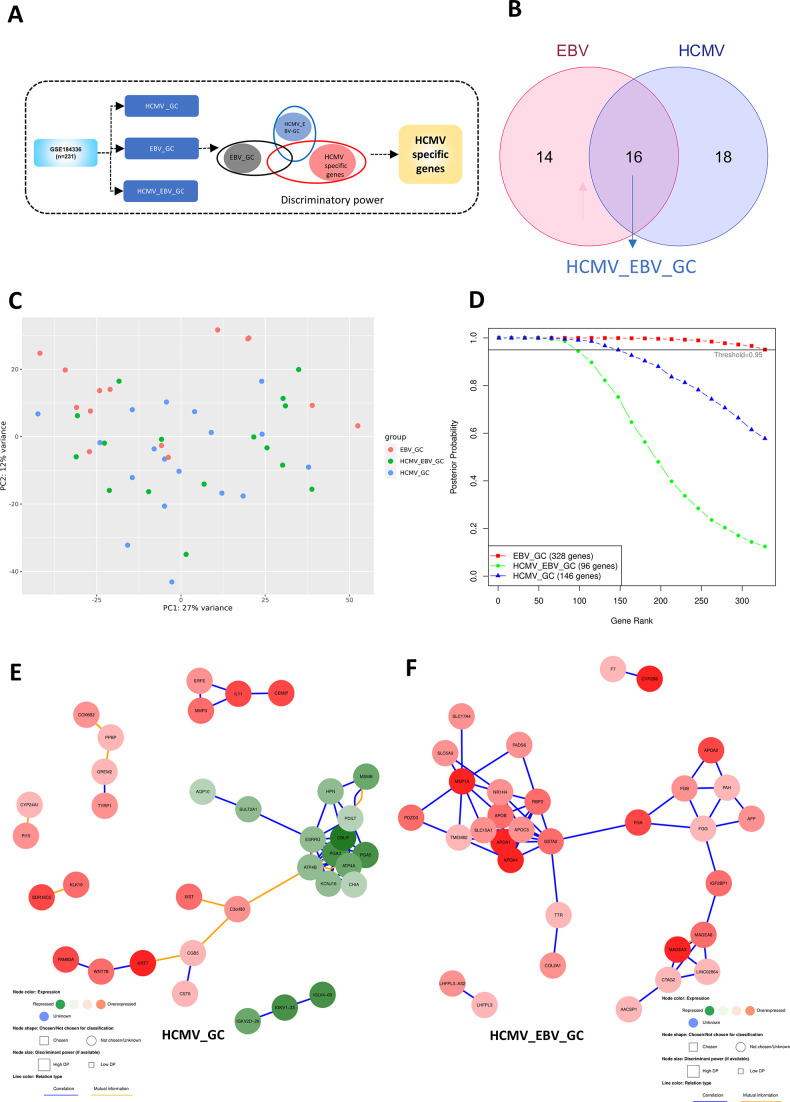
HCMV_GC specific signature identified ranking algorithm based on posterior probability. (**A**) Schematic of the approach implemented to identify the genes that are specifically dysregulated in the HCMV_GC group (minimum of 10 HCMV reads and no EBV reads) compared to HCMV_EBV_GC (presence of HCMV and EBV cumulative reads with the minimum sum of 10 reads) and EBV_GC groups. (**B**) Venn diagram depicting the number of samples in each group. (**C**) The PCA plot depicts the segregation of three groups. (**D**) The genes ranked and considered significant in each class are visualized, setting the posterior probability threshold to be more than 0.95. (**E**) Gene correlation and interaction network of the top 50 genes in the HCMV_GC group and (**F**) the HCMV_EBV_GC group identifies the gene clusters associated with the group.

There can be possible co-infections between HCMV and EBV that can affect each other pathogenesis. Although the involvement of EBV in GC progression has been reported, there are no reports that explore the transcriptome signature specific for the HCMV-positive GC and HCMV_EBV-positive GC patients. For multi-class problem, geNetClassifier algorithm was used to segregate the genes specifically dysregulated in HCMV_GC associated group (Fig. 6A). Three groups were assigned to identify the HCMV-GC-specific gene signatures. Eighteen samples with a minimum of 10 HCMV reads, but no EBV reads were categorized as the HCMV_GC group. Fourteen samples with a minimum of 10 EBV reads, but no HCMV reads were categorized as the EBV_GC group. Sixteen samples with the presence of both EBV and HCMV viral reads with a cumulative number of viral reads to a minimum of 10 reads were categorized as the HCMV_EBV_GC group (Fig. 6B). The raw counts were normalized, and the PCA plot was utilized to observe the segregation of three groups (Fig. 6C). Since the motive is to identify the genes that are dysregulated, specifically in the HCMV_GC group only, the geNetclassifier algorithm is implemented. With a posterior probability cutoff of more than 0.95, 146 genes were found to be specifically associated with the HCMV_GC group and 96 genes to be associated with the HCMV_EBV_GC group, respectively (Fig. 6D), (Table S5). Gene interaction and correlation networks were constructed based on the top 50 genes sorted by the discriminatory power in the HCMV_GC group (Fig. 6E), HCMV_EBV_GC group (Fig. 6F), and EBV_GC group (Fig. S5).

### Gastric acid secretion pathway genes are specifically dysregulated in the HCMV_GC group

**Fig 7:**
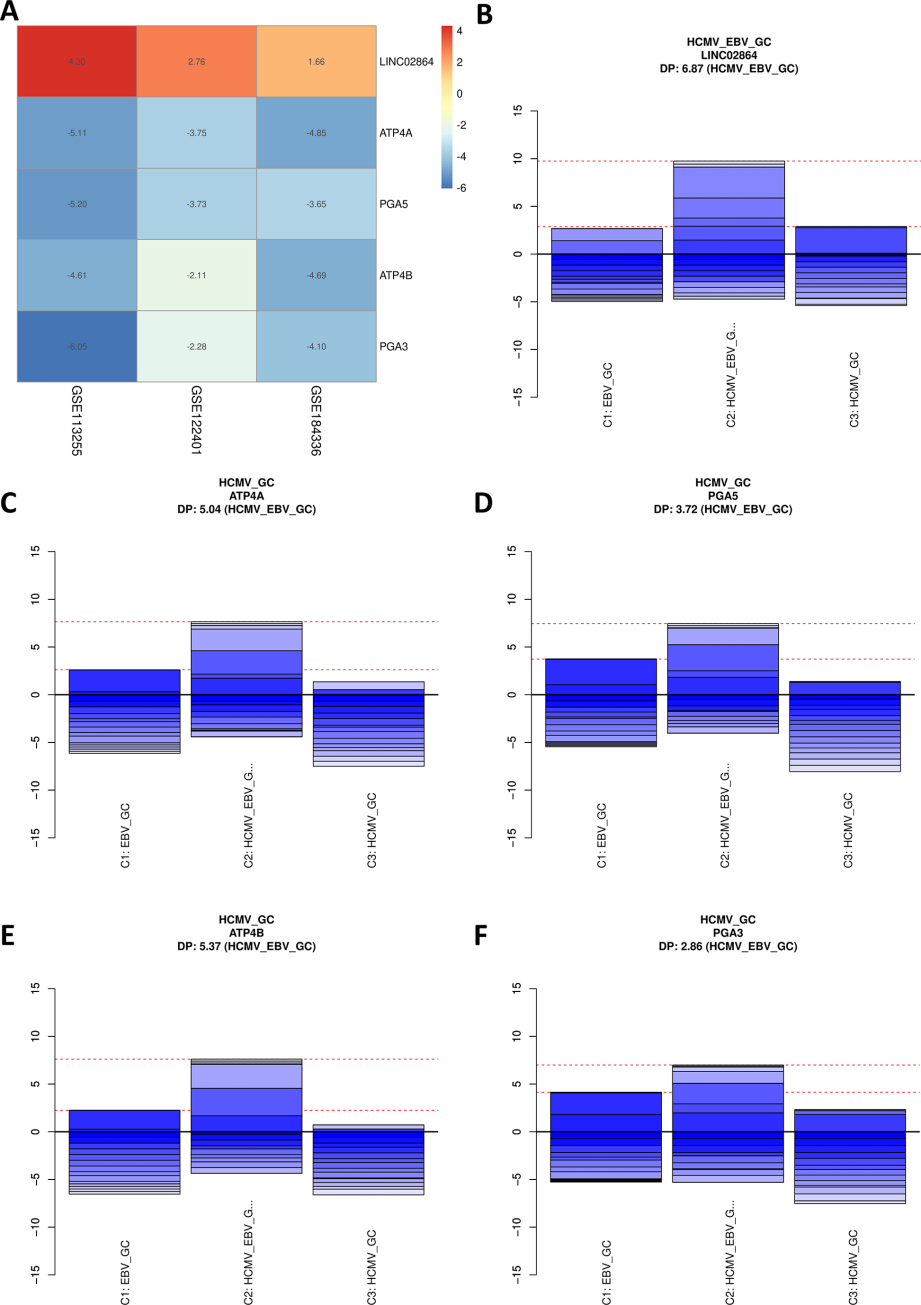
Gastric acid secretion pathway genes are specific for HCMV-specific GC. (**A**) Comparison of the genes identified through rank-based meta-analysis and HCMV_GC specific ranked genes above posterior probability against EBV_GC and HCMV_EBV_GC groups identified gastric acid secretion pathway genes such as ATP4A, PGA5, ATP4B, and PGA3 to be significantly dysregulated by HCMV. (**B**) While LINC02864 is specifically dysregulated in HCMV_EBV_GC group, (**C**) ATP4A, (**D**) PGA5, (**E**) ATP4B, and (**F**) PGA3 were specifically downregulated in HCMV_GC group.

It is observed that the downregulated genes in HCMV_GC groups were involved in the gastric acid secretion pathway, as observed in the meta-analysis (Fig. 7A). Interestingly, LINC02864, found to be dysregulated across all the HCMVPosGC data sets, was specifically upregulated when there was a co-infection of HCMV and EBV (Fig. 7B). The genes that are dysregulated in meta-analyses, such as ATP4A (ATPase H^+^/K^+^ transporting subunit alpha) (Fig. 7C), PGA5 (pepsinogen A5) (Fig. 7D), ATP4B (ATPase H^+^/K^+^ transporting subunit beta) (Fig. 7E), and PGA3 (pepsinogen A3) (Fig. 7F), are very much downregulated only in HCMV_GC, implying that these genes and associated pathways are modulated specifically by HCMV infection. Several reports suggest that these genes act as tumor suppressors in GC, and its downregulation by HCMV suggests that HCMV infection can guide to worse GC outcomes.

### LTF and KLK10 are associated with the progression of HCMV_GC toward later stages

**Fig 8:**
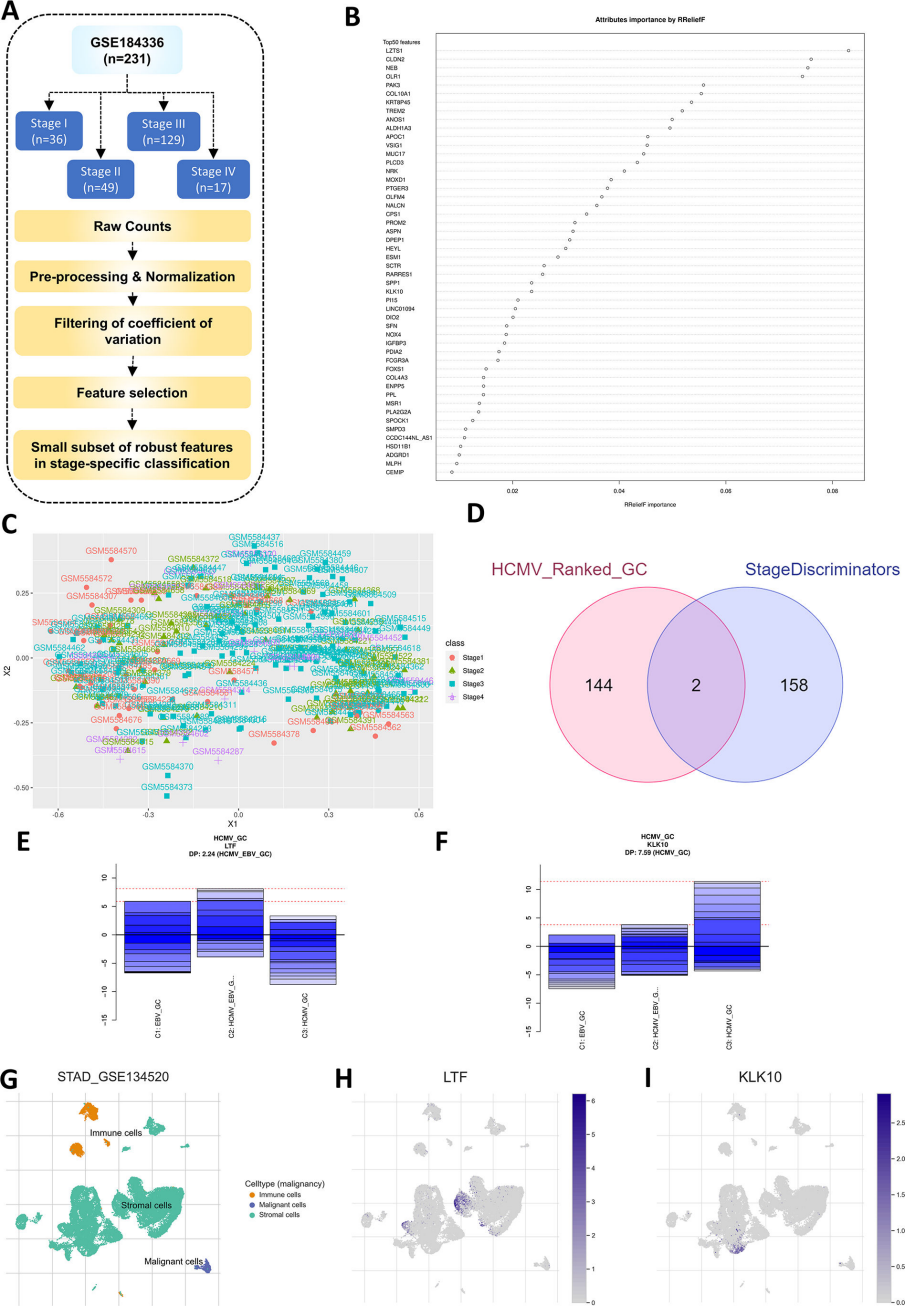
GC stage-wise discriminatory features identify genes associated with HCMV and late stage of GC. (**A**) The GSE184336 data set was segregated into groups with four different stages of GC as provided in metadata and was processed to identify the best set of features that can discriminate different stages of GC. (**B**) Feature selection is performed based on RRelief importance scores, and the top 50 genes are plotted. (**C**) The MDS plot depicts the segregation of samples based on the expression of the 160 genes identified as significant discriminatory features. (**D**) Venn diagram depicting the identified two genes common between ranked HCMV_GC and stage discriminators. (**E**) LTF expression was specifically downregulated, while (**F**) KLK10 expression was specifically upregulated in the HCMV_GC group. (**G**) The single-cell RNA-seq reanalysis of GSE134520 depicts that (**H**) LTF and (**I**) KLK10 expression is predominantly found in GC patients' stromal cells.

The observation that expression of tumor suppressor genes was downregulated in the HCMV_GC group and upregulation of LINC02864 and MAGEA10, whose increased expression is associated with poorer survival, suggest there can be an association of any HCMV-specific genes whose expression changes across different stages of GC progression. GSE184336 data set provided information about the stages of each GC patient, and hence, the best subset of genes that can discriminate between four stages of GC was identified (Fig. 8A). DaMiRSeq method was implemented to identify the subset of genes that have a higher potential to discriminate between different stages of GC through backward variable elimination with a partial least-squares regression approach. One hundred forty genes were identified as having the potential to discriminate between different stages of GC and are sorted based on their RRelief importance score, as explained in the methods (Fig. 8B and C), (Table S6). The cluster plot was plotted to visualize the change in the expression between different stages (Fig. S6). A total of two genes were observed to be common between the HCMV_GC ranked group obtained from the genetclassifier algorithm and stage discriminators (Fig. 8D). These genes are specifically dysregulated in HCMV_GC group and are also indicators of the progression of GC toward later stages.

The expression of lactoferrin (LTF) is suppressed specifically in the HCMV_GC group (Fig. 8E). Kallikrein-related peptidase-10 (KLK10) was upregulated in the HCMV_GC group compared to other groups (Fig. 8F). The expression of these two genes in different cell populations was identified through reanalysis of the single-cell RNA-sequencing data set GSE134520. It was observed that they are mostly expressed in stromal cells and minimal amount in malignant cells, suggesting they can be good prognostic markers for HCMV-positive GC late-stage progression (Fig. 8G through I).

## DISCUSSION

This study utilizes the potential of NGS to detect the presence of virus reads in the Asian GC clinical samples to identify the detection of EBV and HCMV. The existing studies screening for virus reads in the GC samples are mostly from the data sets derived from TCGA that contain a limited number of GC samples from Asian ancestry. Nearly three out of four GC cases reported globally are from Asia. The RNA-seq data sets used in this study were derived from a large sample size of GC tumor samples from China and Korea by three independent groups, reducing the possibility of false-positive results or results due to technical artifacts obtained from a single study. Three different approaches including screening of viruses that have minimum of 10 reads in minimum 10 samples of each data set (Fig. 2A), viruses with higher mean value of virus reads aligned per human reads aligned to the genome (Fig. 2B through D) and screening for viruses with cumulative reads more than 1,000 in each sample, identified the higher presence of EBV and HCMV genomes in all three data sets (Fig. 2E through G). Although EBV presence in GC samples is well-known, the presence of HCMV in GC is not well explored in GC pathogenesis.

HCMV produces four known long non-coding RNA transcripts namely RNA2.7, RNA1.2, RNA4.9, and RNA5.0. RNA 2.7 is located between the locus of the genes RL1 and RL5A and has been reported to enhance the viral spread in lytic infection by regulating the cell motility (26) and it has been detected across all the HCMVPosGC patients in all three data sets (Fig. S3). RNA1.2 is located between the locus of the genes RL6 and RL8A and has been reported to play an important role in modulating intrinsic NF-κB-dependent signaling and thereby regulating downstream immune responses (27). RNA4.9 is located near the UL69 gene and it is one of the well-characterized to be playing an important role in HCMV replication (28). RNA5.0 is located between the locus of the genes UL105 and UL111A, is a stable intron and its function in HCMV pathogenesis is not well characterized. In our analyses, RNA2.7 and RNA1.2 are detected in most of the samples across all three data sets, while RNA4.9 and RNA5.0 are detected in few samples only (Fig. S3).

Although the main purpose of this study is to detect the virome in GC samples, it is important not to exclude the detection of *H. pylori*, bacteria that is well-known to be associated with GC progression. *H. pylori* is detected in 200 out of 230 samples, 125 out of 130 samples, and 80 out of 80 samples in the GSE184336, GSE113255, and GSE122401 data sets, respectively (Fig. S7A and B). The observation of *H. pylori* in most of the GC samples is not surprising as the prevalence of *H. pylori* in GC cases was already reported to be high. Out of 3,161 GC cases involved in a study in *H. pylori*-negative Japan, only 21 cases were regarded as *H. pylori*-negative in a study (29). Another Korean study identified only 28 samples as *H. pylori*-negative out of 75 GC cases included in the study (30). It is important to note that both HCMV-positive and HCMV-negative groups consist of *H. pylori* presence equally. Out of 22 samples considered negative for *H. pylori*-negative in (Fig. S7B), 7 samples have less than 10 reads of EBV or HCMV and hence discarded for further analysis (Table S7). Differential expression analysis between *H. pylori*-negative GC (15) group that comprised samples that had no reads for *H. pylori*, HCMV, and EBV and *H. pylori*-positive group (141) that had minimum of 10 *H. pylori* reads but no reads detected for EBV and HCMV identified genes that are not related to the genes identified specific for HCMV (Table S7). Hence, the influence of *H. pylori* in HCMV-specific signature is minimal (Fig. S7C and D).

To further validate the bioinformatics findings of the occurrence of HCMV, GC patients were recruited from Mizoram, India. Using the nested PCR, 6 out of 42 GC samples were tested positive for HCMV. This is also the first study that explores the presence of viruses in GC samples derived from India. It was found that there are no previous reports that explored the host transcriptome alterations due to the presence of HCMV in GC samples. Hence, differential expression analysis was performed to identify the genes dysregulated in the HCMVPosGC. Robust parameters, such as a minimum of 10 HCMV reads, were used to assign samples in the HCMVPosGC group. To avoid the possible interference of EBV, the HCMVNegGC group consists of GC samples with no reads of both EBV and HCMV. For the identification of robust genes that are dysregulated, the robust rank aggregation meta-analysis was performed. This is to ensure that the genes found significant were consistently dysregulated in all three independent data sets. The gastric acid secretion pathway was found to be significantly altered by HCMVPosGC. In addition to this, LINC02864 and MAGEA10 gene expression was consistently upregulated in HCMVPosGC. Both these genes were oncogenes, and downregulated genes such as ATP4A, ATP4B, PGA3, PGA4, and PGA5 were reported tumor suppressors in GC. Since the tumor suppressor genes are downregulated and oncogenic genes are upregulated, HCMV infection may be associated with the increased progression of GC.

EBV and HCMV co-infection and their role in GC pathogenesis is unexplored. It is important to identify the HCMV-specific genes that are different from EBV alone or EBV and HCMV co-infected patients. geNetClassifier ranks each gene based on an expectation-maximization (EM) algorithm that compares the differential expression between all the groups and reports the posterior probability of each gene belonging to a particular class. This approach is suitable for multi-class classification. One hundred forty-six genes that are significantly associated with only the HCMV_GC group were identified. The correlation network constructed based on the top 50 genes identified the cluster of genes belonging to the gastric acid secretion pathway. Interestingly, LINC02864 and MAGEA family proteins were upregulated in HCMV and EBV co-infected patients. One bioinformatics study predicted that LINC02864 overexpression is associated with poorer survival outcomes in GC patients and might be associated with *H. pylori* pathogenesis (31). However, this *in silico* study identified LINC02864 (RP11-169F17.1) to be significantly dysregulated between normal and stomach adenocarcinoma (STAD) tumor samples in differential expression analysis. Further, LncRNA-miRNA target enrichment analysis predicted STAD dysregulated miRNAs that could be regulated by this LncRNA. miRNA target enrichment analysis identified the genes that overlapped with *H. pylori*-associated gene signatures. So the authors of this study suspected that this lncRNA could be associated with *H. pylori* infection, as it is predicted to target miRNAs that are predicted to target *H. pylori*-specific genes such as BCL2, MYC, E2F3, CCND1, APC and TWIST1. It is important to note that these genes are not only specific to *H. pylori* but also play a crucial role in HCMV and EBV pathogenesis. The differential expression analysis between *H. pylori-*negative GC and *H. pylori*-positive GC in our study did not identify LINC02864 to be significantly dysregulated. Future studies are needed to characterize this long non-coding RNA and its association with GC progression. MAGEA family proteins were also reported to be associated with GC progression (32), and how HCMV infection utilizes this protein also needs to be explored further. ATP4A, ATP4B, PGA3, and PGA5 were reported to be early-stage GC biomarkers and their suppression is associated with poorer survival (33). Their abrupt downregulation in the HCMV_GC group implies that HCMV infection may guide the progression of GC.

Using the feature selection approach implemented in the DamiRSeq machine learning pipeline, 160 genes that can discriminate between different stages of GC were identified. Among these, LTF and KLK10 were associated with HCMV_GC also. LTF is shown to have anti-cancer activity in GC and their therapeutic potential in GC treatment was explored in a study using LTF-coated iron nanospheres (34). Interestingly, LTF can inhibit HCMV replication in stromal cells (35). It can be speculated that decreased expression of LTF in the HCMV_GC group may be an indication of GC progression. KLK10 expression was upregulated specifically in the HCMV_GC group and was reported to be an indicator of the incurability of GC (36). Both these genes were identified to be expressed mostly in the stromal cell population using single-cell RNA-seq data of GC patients.

This study also has room for improvement. The motive of our study is to identify the presence of viral genomes in the GC samples derived from Asian ancestry and since HCMV presence was evident in the three independent data sets using three different screening approaches, the next aim was to find the host transcriptome dysregulation in these samples. However, our study is limited to the detection of HCMV genome but not which types of cells the HCMV viral reads are distributed and which type of non-coding RNA presence. The quantification of HCMV in single-cell RNA-seq data sets of Asian GC patients will be the more appropriate method to identify the viral reads in single-cell resolution. However, the existing scRNA-seq data sets do not have enough GC patients to come up with conclusion. Future plan is to explore the HCMV identification in the single-cell level and the exact characterization of which type of RNA transcripts derived from the HCMV genome warrants biochemical and molecular techniques that will be the part of future studies. The initial part of the study that quantified the virus reads from Asian GC samples through the Bioinformatics pipeline was based on the available metadata information. Future studies should explore more information, such as the stage of HCMV latency, *H. pylori* infection status, and treatment/therapy information in a large cohort. Future studies can explore the infection status of HCMV in GC and measure pepsinogen levels and the correlation between HCMV viral load. It will be more interesting to explore the presence of HCMV, and EBV DNA in the blood samples that can be utilized for diagnosis for better GC risk stratification.

### Conclusions

Apart from the well-studied association between *H. pylori* and EBV, this study identified the presence of HCMV in Asian GC samples and the associated gene signature supporting the progressive role of HCMV in GC. The observation of the involvement of gastric acid secretion pathway, LINC02864, MAGEA family proteins, LTF, and KLK10 with HCMV infection will guide future studies to explore the previously undermined involvement of HCMV in the GC pathogenesis and early identification of high-risk GC patients based on the presence of HCMV infection.

## SUPPLEMENTAL MATERIAL

Table S1 msystems.00673-24-s0001.docx Indian GC cohort demographic details.

Table S2 msystems.00673-24-s0002.xlsx Details of the samples found to be HCMVPosGC and HCMVNegGC in the three data sets.

Supplemental Figures msystems.00673-24-s0003.pdf Figures S1 to S7.

Legends msystems.00673-24-s0004.docx Legends for supplemental figures and tables.

Supplemental Tables msystems.00673-24-s0005.xlsx Tables S3 to S7.

ASM does not own the copyrights to Supplemental Material that may be linked to, or accessed through, an article. The authors have granted ASM a non-exclusive, world-wide license to publish the Supplemental Material files. Please contact the corresponding author directly for reuse.
