# Supplementary material for: HCMV detection in Asian gastric cancer RNA-seq data sets and clinical validation in Indian GC patients reveals the HCMV-GC specific gene signatures
Source: mSystems. 2024 Sep 16;9(10):e00673-24. doi: 10.1128/msystems.00673-24 (PMC11494955; doi:10.1128/msystems.00673-24)
Supplement: Table S1 — Indian GC cohort demographic details. [file msystems.00673-24-s0001.docx]

**Table S1: Indian GC cohort demographic details.**

| **Sl.No.** | **Sample ID** | **Sex** | **Age** (years) | **Surgery type** | **Stage / Grade** |
| --- | --- | --- | --- | --- | --- |
| 1. | GC80T | Male | 53 | Total gastrectomy | T3 N0 |
| 2. | GC81T | Male | 60 | Subtotal gastrectomy | T4a N0 |
| 3. | GC82T | Female | 69 | Subtotal gastrectomy | T3 Mx N0 |
| 4. | GC83T | Male | 61 | Subtotal gastrectomy | T2 N2 |
| 5. | GC84T | Female | 65 | Subtotal gastrectomy | T4a N3a |
| 6. | GC85T | Male | 65 | Subtotal gastrectomy | T2 N0 |
| 7. | GC86T | Male | 45 | Subtotal gastrectomy | T4a N3a |
| 8. | GC87T | Male | 78 | Total gastrectomy | T3 Mx N0 |
| 9. | GC88T | Male | 70 | Subtotal gastrectomy | T2 N0 |
| 10. | GC89T | Female | 43 | Total gastrectomy | NA |
| 11. | GC90T | Female | 60 | Subtotal gastrectomy | NA |
| 12. | GC91T | Male | 48 | Subtotal gastrectomy | T3 Mx N2 |
| 13. | GC92T | Male | 50 | Subtotal gastrectomy | T3 N3 |
| 14. | GC93T | Male | 65 | Subtotal gastrectomy | T3 N3b |
| 15. | GC94T | Female | 60 | Total gastrectomy | T4a N3a |
| 16. | GC95T | Female | 56 | Total gastrectomy | T1b N0 |
| 17. | GC96T | Male | 45 | Total gastrectomy | NA |
| 18. | GC97T | Male | 71 | Subtotal gastrectomy | NA |
| 19. | GC98T | Male | 66 | Subtotal gastrectomy | T3 N1 |
| 20. | GC99T | Male | 72 | Subtotal gastrectomy | T3 N3a |
| 21. | GC100T | Male | 67 | Oesophago-total gastrectomy | T4b N3b |
| 22. | GC101T | Male | 55 | Subtotal gastrectomy | T2 N1 |
| 23. | GC102T | Male | 50 | Subtotal gastrectomy | T4a N3b |
| 24. | GC103T | Female | 57 | Oesophago- gastrectomy | T1b N0 |
| 25. | GC104T | Male | 59 | Total gastrectomy | T4a N3b |
| 26. | GC105T | Male | 69 | Subtotal gastrectomy | T3 N3a |
| 27. | GC106T | Male | 69 | Total gastrectomy | T4a N3a |
| 28. | GC107T | Male | 60 | Subtotal gastrectomy | T3 N2 |
| 29. | GC108T | Male | 67 | Subtotal gastrectomy | T2 N0 |
| 30 | GC109T | Male | 67 | Subtotal gastrectomy | T4a N2Mx |
| 31 | C110T | Male | 54 | Subtotal gastrectomy | T4a N3a Mx |
| 32 | GC111T | Male | 48 | Subtotal gastrectomy | T4a N2 |
| 33 | GC112T | Female | 67 | Subtotal gastrectomy | T4b N0 |
| 34 | GC113T | Female | 48 | Subtotal gastrectomy | T4b N3 |
| 35 | GC114T | Male | 74 | Subtotal gastrectomy | T3 N2 |
| 36 | GC115T | Female | 54 | Subtotal gastrectomy | T3 N2 |
| 37 | GC116T | Male | 65 | Subtotal gastrectomy | T4a N3a |
| 38 | GC117T | Male | 57 | Distal gastrectomy | T2 N0 |
| 39 | GC118T | Male | 58 | Subtotal gastrectomy | T3 N3a |
| 40 | GC119T | Male | 65 | Total gastrectomy | T3 N0 |
| 41 | GC120T | Male | 57 | Distal gastrectomy | NA |
| 42 | GC121T | Male | 72 | Subtotal gastrectomy | NA |
