# Supplementary material for: HCMV detection in Asian gastric cancer RNA-seq data sets and clinical validation in Indian GC patients reveals the HCMV-GC specific gene signatures
Source: mSystems. 2024 Sep 16;9(10):e00673-24. doi: 10.1128/msystems.00673-24 (PMC11494955; doi:10.1128/msystems.00673-24)

Fig. S1: PRISMA Guidelines to identify Asian GC patients.

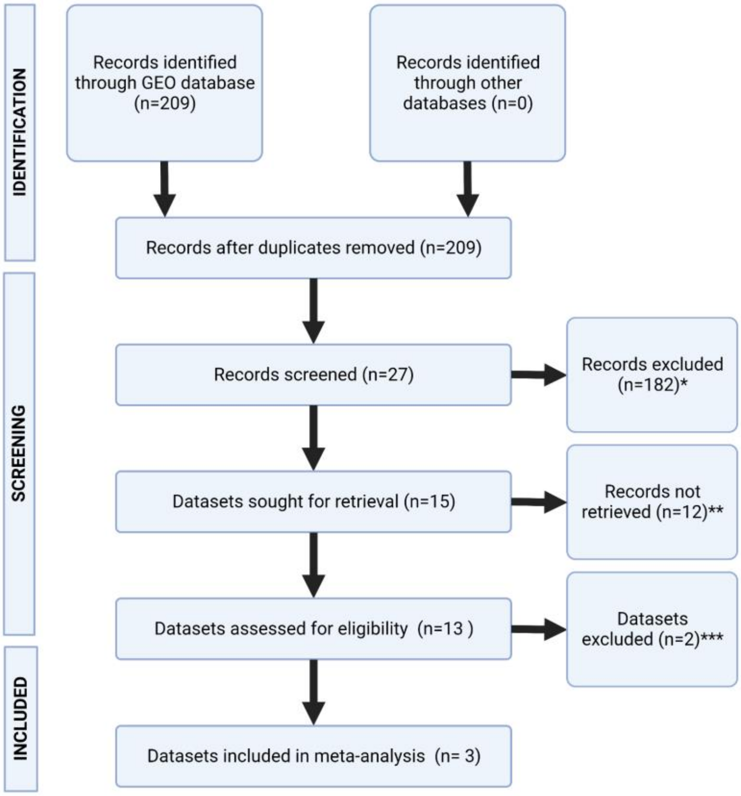

GSE ID	Ancestry	Origin	No. of samples
GSE184336	Asian	China	231
GSE113255	Asian	Korea	130
GSE122401	Asian	Korea	80

*Datasets less than 50 samples per group are excluded.
**Datasets other than gene expression profiling are excluded.
***Datasets other than samples of Asian origin and paired end reads are excluded.

Fig. S2 : The Venn diagrams representing the top viruses detected in the datasets (A) GSE184336, (B) GSE113255 and (C) GSE122401. (D) Bubble plot denotes the number of reads assigned to different viruses where the size of the dots represents the number of reads and the red color indicates GSE113255, Blue indicates GSE113255 and Green indicates GSE184336, from which the samples are derived.

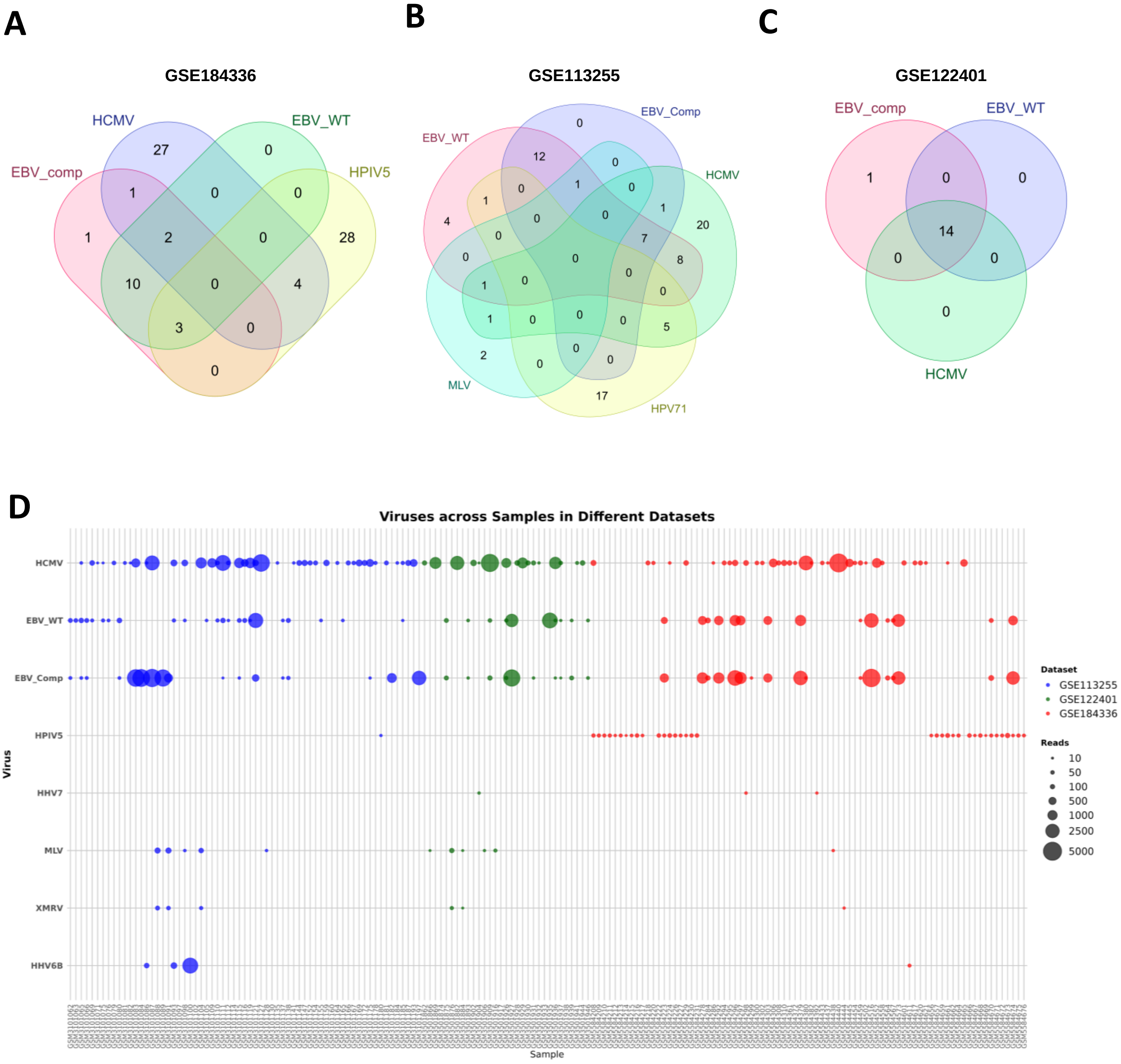

Fig. S3: The coverage plot of four major non-coding RNAs of HCMV in HCMVPosGc group patients in GSE184336, GSE113255 and GSE124401 represented in red, green and blue respectively.

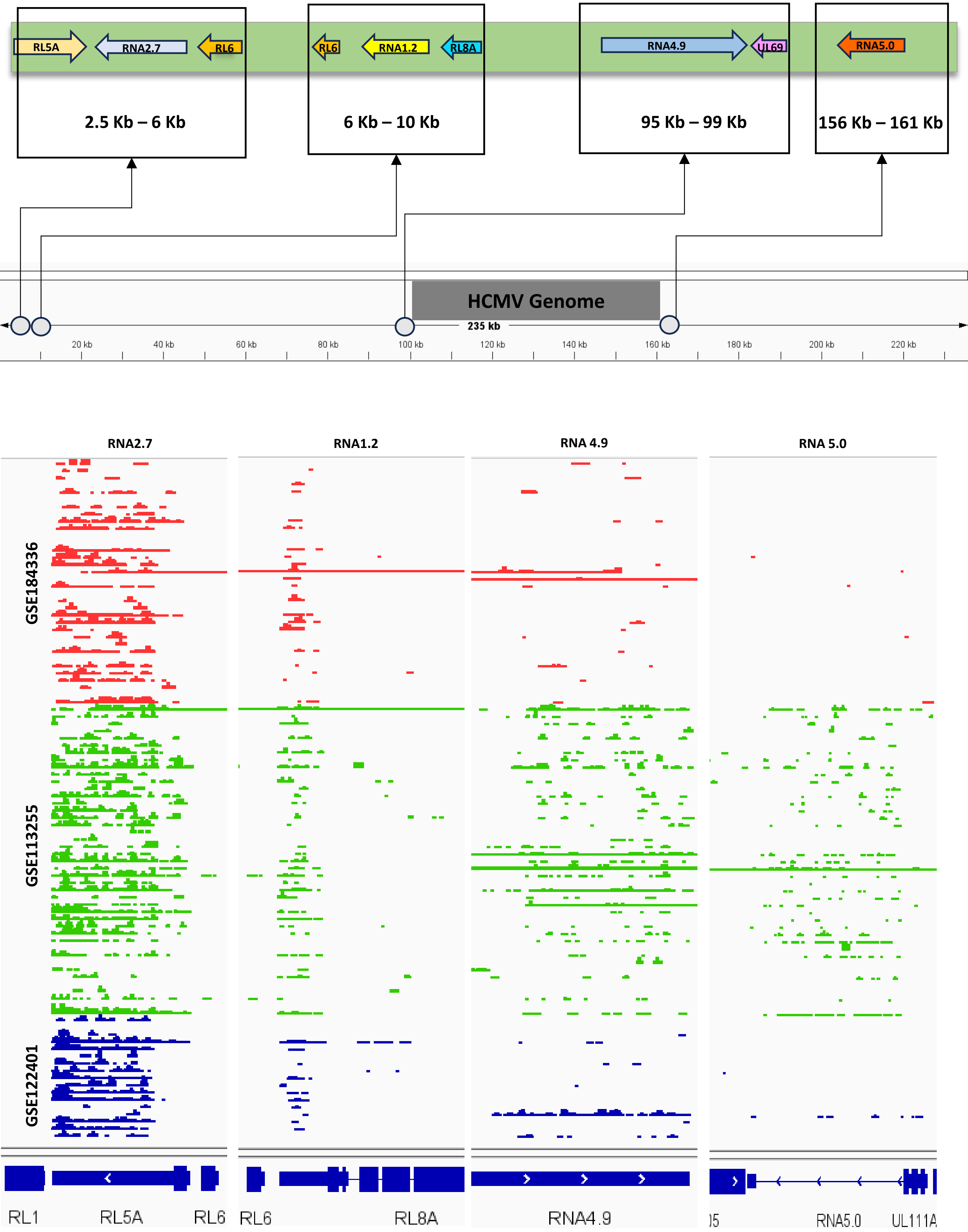

Fig. S4: Schematic of rank-based meta-analysis and differential expression analysis results visualized in volcano plots for GSE113255 and GSE122401.

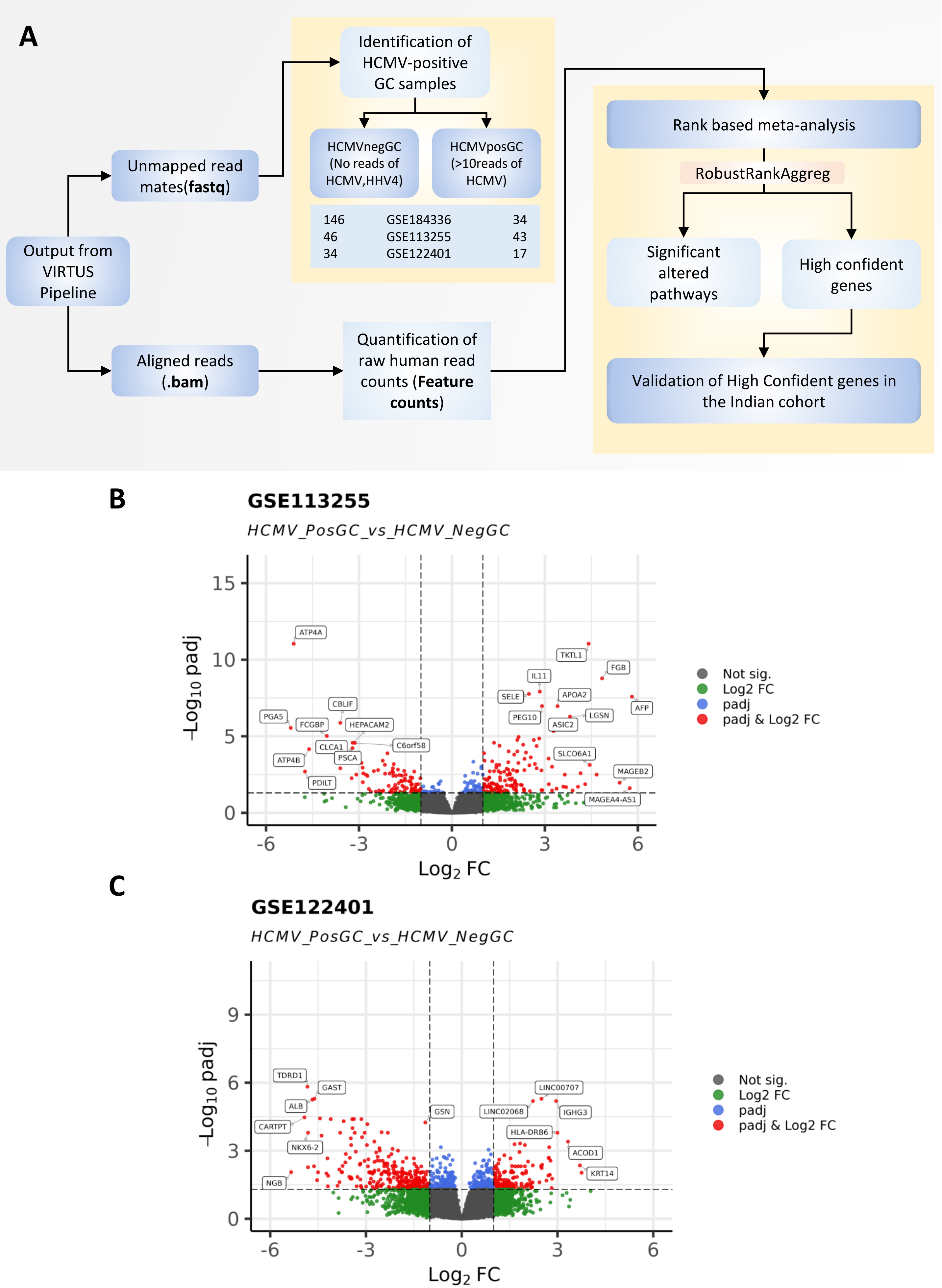

Fig. S5: The gene correlation network plotted for the EBV_GC specific genes identified through the geNetClassifier approach.

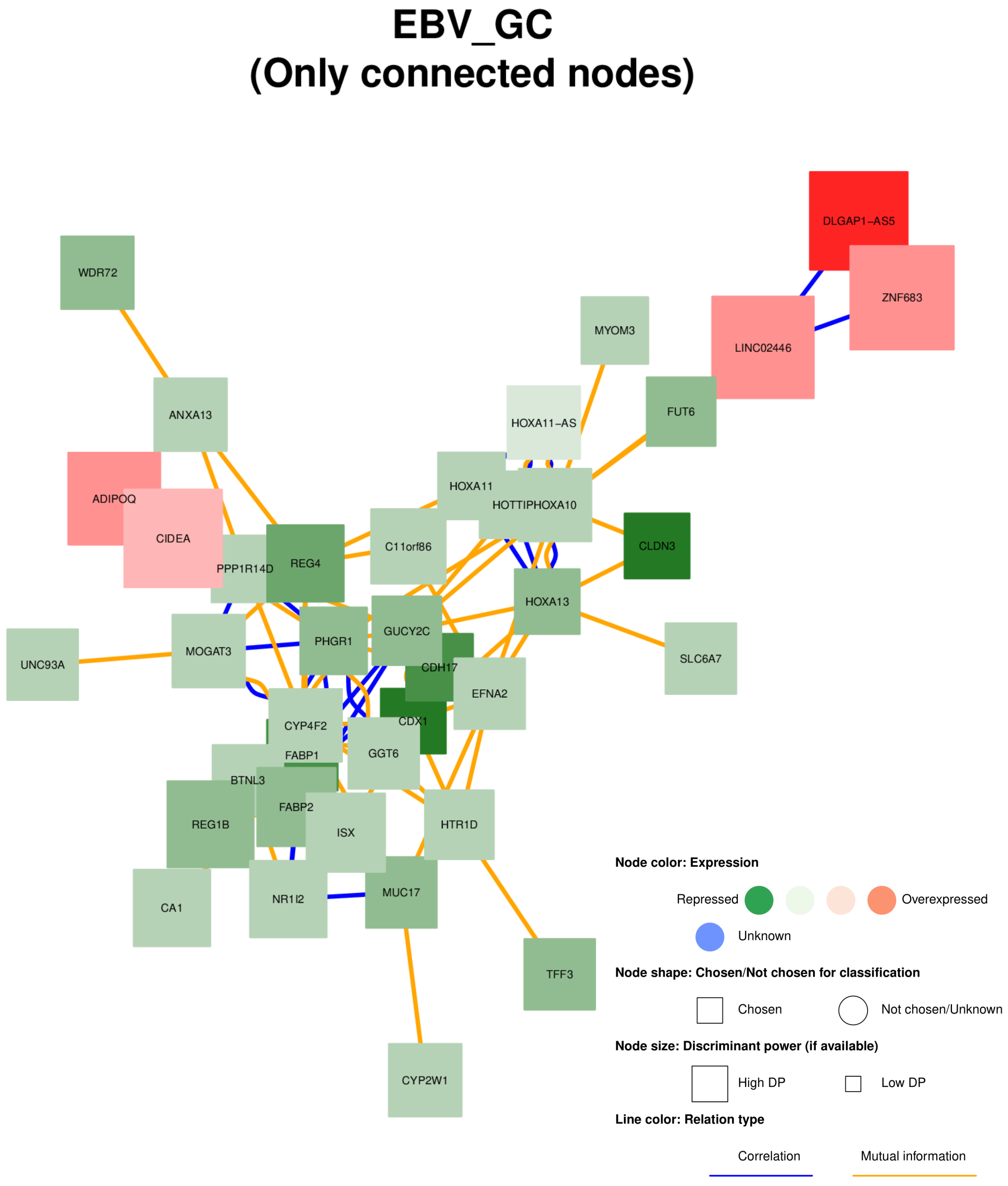

Fig. S6: Cluster plot showing the expression of top discriminatory genes for GC stages.

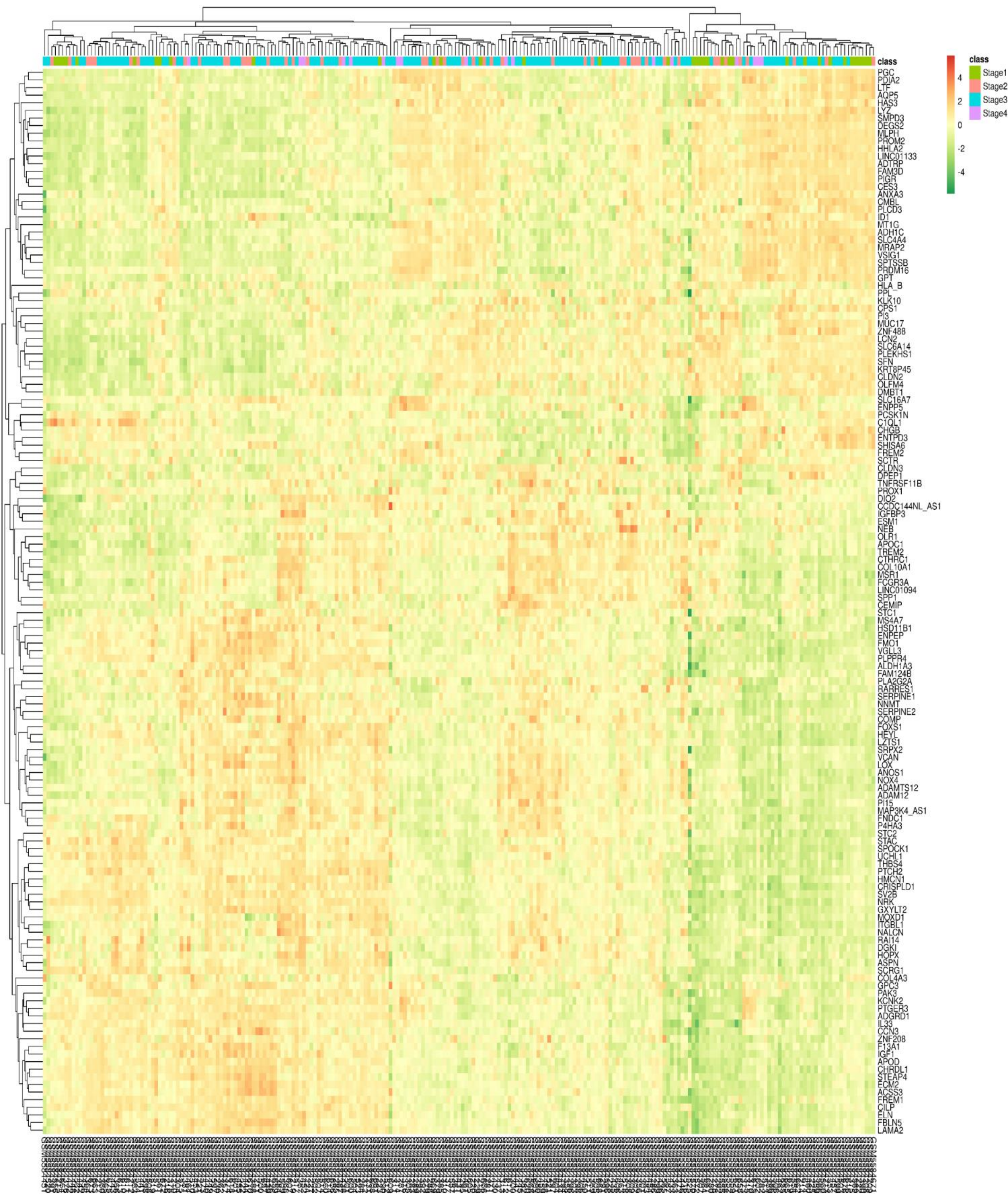

Fig. S7: Detection of *H.pylori* reads in GC samples.(A) The dot plot representing the presence (green) and absence (red) of *H.pylori* in the samples belonging to three datasets and the bubble size corresponds to total number of reads in each sample.(B)Venn diagram represents the detection and correlation of H.pylori with EBV and HCMV in the dataset GSE184336. Please note that all the samples in GSE113255 are positive for *H.pylori*.(C) Differential expression analysis was performed between 22 H.pylori, EBV and HCMV negative samples and H.pylori positive but EBV and HCMV negative samples. PCA plot depicts the segregation between the two groups.(D)The HCMV_GC rank based meta-analysis genes and the differentially expressed genes in H,pyloripos vs H.pyloriNeg were plotted in venn diagram. There were no common genes and hence it is interpreted that HCMV signature identified in the study is specific to HCMV.

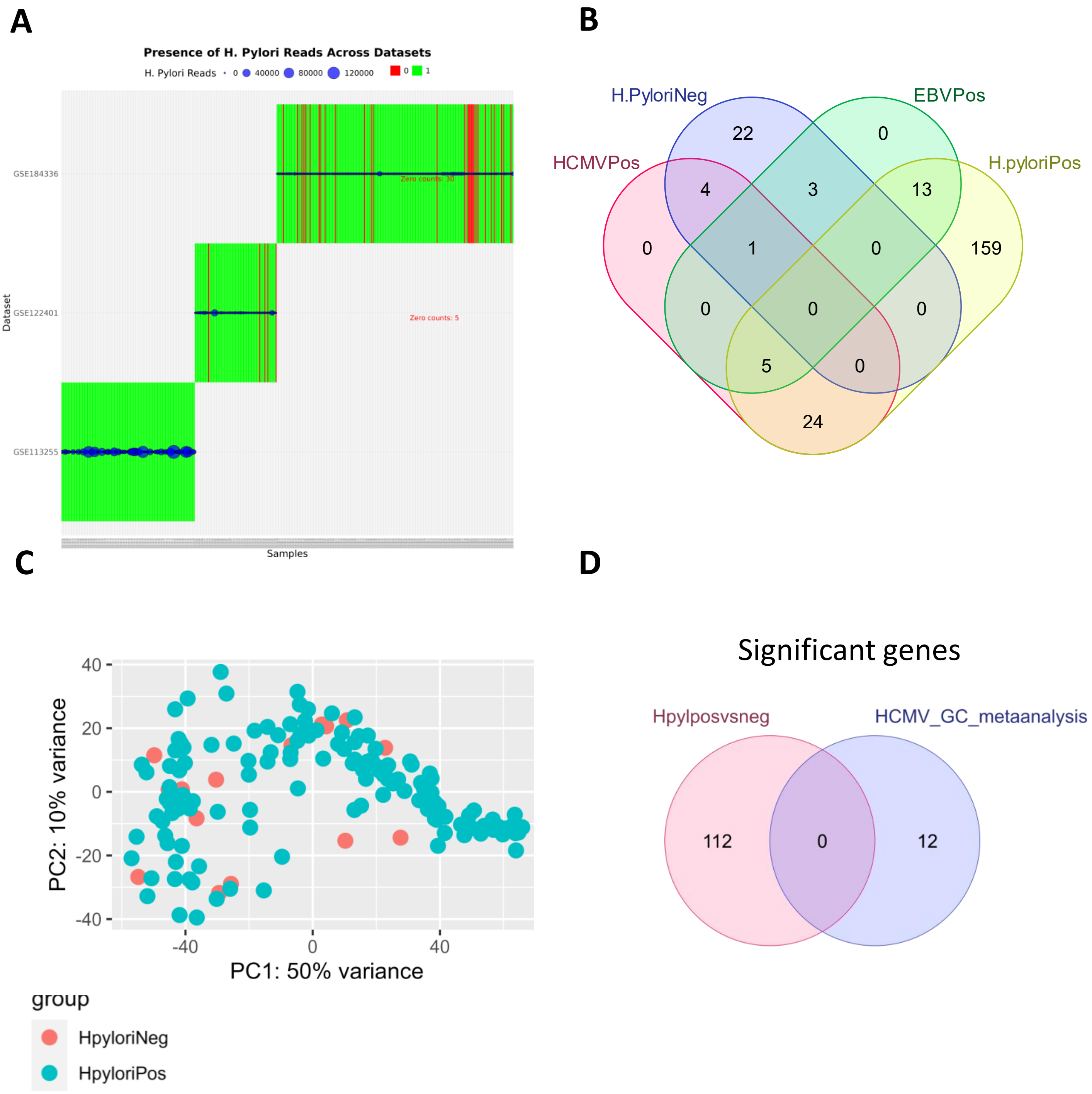

Supplement: Supplemental Figures — Figures S1 to S7. [file msystems.00673-24-s0003.pdf]
